# Publishing in English or Chinese: a qualitative analysis of Chinese researchers’ academic language choice

**DOI:** 10.3389/fpsyg.2023.1249857

**Published:** 2023-09-20

**Authors:** Jing Cui, Changbo Qiu, Zhigang Wang

**Affiliations:** School of Business and Management, Jilin University, Changchun, China

**Keywords:** journal attributes, academic language choice, grounded theory, non-native language, Chinese researchers

## Abstract

Non-native language scholars often struggle to choose between English and their native language in scholarly publishing. This study aims to identify the mechanism by which journal attributes influence language choice by investigating the perspectives of 18 Chinese scholars through semi-structured interviews. Drawing on grounded theory, this study develops a model for how journal attributes influence researchers’ language preferences. We find that journal attributes influence researchers’ perceived value which, in turn, affects their particular language choice, with contextual factors playing a moderating role. By examining the motivations underlying Chinese scholars’ language choice, this study provides a critical understanding of the factors shaping their decision-making processes. These findings have significant implications for Chinese scholars, policymakers, and journal operators, shedding light on the issue of discrimination in academic publishing. Addressing these concerns is crucial for fostering a fair and inclusive academic environment.

## 1. Introduction

English plays an extremely important role in the academic world; almost all international academic communication takes place in English. Moreover, English is the predominant language used for writing and publishing a large body of academic research and literature. Publications in languages other than English suffer from discrimination when their scientific output is evaluated based on the number of citations in citation databases (Towpik, 2015; López-Navarro et al., 2015b; Furnham, 2020).

Natural science fields and university settings are becoming increasingly globalized, and to reach the greatest number of their fellow academics, scholars face pressure to publish their work in the most widely used language. In contrast, those employed in non-academic settings are more likely to aim to engage local policymakers and audiences (Gingras, 1984; Stockemer and Wigginton, 2019). Furthermore, the greater use of English by younger scholars and possible increases in prestige incentives may increase its dominance over time. This applies even more when considering the push to embrace the dominance of English (Lublin, 2018).

The large number of papers published in international journals, i.e., English-language academic papers, is detrimental not only to the local dissemination of academic research but also to the development of native-language publications (Kuteeva and Mauranen, 2014). Accordingly, it is important to ask: why do non-native English-speaking scholars choose to publish in English despite the linguistic difficulties it implies? What factors influence their academic language choice? To reduce the negative impacts of writing in English, it is particularly important to understand the mechanisms underlying Chinese researchers’ academic language choice.

Scholars, especially those from non-English-speaking countries, have attached great importance to the study of academic language. However, existing studies have mainly focused on analyzing the motivation of academic language choice (Cho, 2004; Curry and Lillis, 2004; Huang, 2011; Martín et al., 2014; Mu and Lawrence, 2018) and the influence of articles in different languages (Li et al., 2014 Liu, 2017). Several scholars have highlighted how the difficulty of mastering English and the preference for writing in the style of native speakers limit the ability of non-native speakers to produce and consume academic literature (Benfield and Howard, 2000; Flowerdew, 2001, 2008, 2012; Braine, 2002; Flowerdew and Li, 2009). Others raise concerns that the use of English may privilege Western social networks and cultural norms and thus further limit the participation of scholars from developing nations (Canagarajah, 1996, 1999 Lillis and Curry, 2010). Although related studies have analyzed the factors influencing academic language choice from different perspectives, the structural relationship between the influencing factors has not been sufficiently studied, and the mechanism of academic language choice lacks systematic analysis.

Therefore, this study aims to explore the mechanisms behind choosing the language of academic papers through respondents’ perceptions of journal attributes, based on grounded theory analysis. Through this study, we further investigate the influencing factors and their structural relationships, providing relevant strategies for policymakers to encourage more researchers to publish articles in different languages.

## 2. Literature review

The factors that influence authors’ choice of journal can be divided into three categories: author attributes, journal attributes, and other research attributes (Björk, and Holmström, 2006; Cheung, 2008; Knight and Theresa, 2008; Björk and Öörni, 2009). Author attributes include author evaluations of journals, authors’ successful submission experiences, and so on. Journal attributes include the quality of the review process, publication delay, risk of rejection, journal services, technical features, fees, local reputation, professionalism, impact, credibility, international reputation, and likelihood of acceptance (Pepermans and Sandra, 2016). Other research attributes include the potential impact of the literature, communication strategies, and ethical issues. Through an online survey of 5,500 authors, Rowlands and Nicholas (2005) found that the most important factors influencing authors’ choice of journals are journal reputation, audience, and journal impact factor. Tenopir et al. (2016) found, through a survey of 2,021 researchers, that the most influential attributes for journal selection are journal quality and reputation, as well as a good fit with the journal’s research theme. Clearly, journal attributes play a decisive role in authors’ choice of journals (Rousseau and Ronald, 2012). However, there is little current research on the influence of journal attributes on researchers’ language choice for their papers, though the papers cited above provide some stimulating insights.

Currently, research on the choice of language for academic papers mainly focuses on the motivations or attitudes of non-native English-speaking researchers toward language selection. From the perspective of expectancy-value theory, scholars have studied the impact of perceived usefulness, intrinsic value, self-efficacy, and the cost of publishing in a particular language on language selection (Lillis and Curry, 2010; Lin et al., 2014). Others have studied the influence of external and internal motivations on language selection from a social psychology perspective (Duszak and Lewkowicz, 2008; Lee and Lee, 2013; López-Navarro et al., 2015a). Previous studies have mainly focused on aspects of researchers’ language proficiency, including their English proficiency (López-Navarro et al., 2015b). However, researchers later found that the choice of language for academic papers is not limited to a binary classification of local language versus non-local language (Ferguson et al., 2011; Flowerdew, 2013; Kuteeva and Mauranen, 2014), but is also influenced by social factors such as publishing experience, academic qualifications, social recognition, and national policies (Elmalik and Nesi, 2008; Uysal, 2014; Işık-Taş, 2018).

Overall, non-native English-speaking scholars are influenced by various factors when choosing between English and their native languages for publication. There is also a range of “ecological variables” such as country, institution, and disciplinary background that can affect language choice (Baldauf, 2001). The majority of qualitative research has predominantly concentrated on studies of motivation. While these studies have greatly enhanced our understanding of language choice for publication, they have not yet effectively revealed broad paradigms of influence or demonstrated how different factors may impact language choice.

## 3. Methodology

### 3.1. Approach

Grounded theory is an exploratory research method proposed by Glaser Barney and Strauss (1967) that allows researchers to provide a theoretical description of the general features of a topic based on empirical observations or data (Martin and Barry, 1986; Lopez-Gonzalez et al., 2018). This bottom-up approach to developing a systematic theory is particularly suitable for addressing research questions that are limited in scope and require theoretical construction.

The main reason for choosing grounded theory as the research method is that relevant theoretical research on the willingness, concepts, and mechanisms of language choice for publication has not yet matured. Therefore, this study adopted grounded theory, initially based on a literature review and theoretical sampling, to extract judgments and perceptions regarding the factors influencing publication language choice. Subsequently, we constructed a theoretical model framework based on the original data, as per the grounded theory approach.

### 3.2. Instrument

The main instrument used for data collection was in-depth interviews. First, a semi-structured interview guide with open-ended items was created based on a literature review and case studies. Second, two research professors with interview experience were invited to assess our interview guide questions. The interview questions were then improved and changed in response to the expert panel feedback and suggestions. The modified interview guide questions were as follows: (1) Why did you choose to publish some of your papers in English? (2) How did you begin working on an English-language paper? (3) What difficulties did you face when writing and publishing in English? (4) Why did you choose to publish some of your papers in Chinese? (5) What difficulties were encountered in writing and publishing in Chinese? (6) What types of emotional differences exist between writing papers in Chinese and English? (7) What language would you prefer to use when publishing your next paper?

### 3.3. Information collection

Eighteen participants were recruited between September and November 2022. They comprised 11 women and 7 men who had all published papers in both English and Chinese in the past 5 years. The interviewees were Ph.D. teachers with research interests covering science and the humanities. Their ages ranged from their mid-twenties to mid-thirties, with an average age of 28 years. The interviewees had diverse professional backgrounds, were amid a career upswing, and had been highly productive in scientific research in the last 5 years. Hence, the interviewees were in a good position to provide valuable insights into the topic under investigation.

Personal in-depth interviews were conducted with all 18 interviewees, each lasting at least 30 min. During the one-on-one interviews, interviewees were given sufficient time to reflect before responding, and interviewers could adjust the questions to uncover deeper influencing factors, thereby gaining a more comprehensive understanding of the attributes that affect their language choices. With participants’ consent, the interview content was recorded, archived, and later transcribed. Fifteen randomly selected interview transcripts were coded, while the remaining three were used to test for saturation. Table 1 presents the interviewees’ basic information.

**Table 1:** Interviewees’ demographic information.

Interviewee	Gender	Age	Major	Recent 5-year Research Achievements	
				ENG	CHN
M1	Male	24	Management Science	4	1
M2	Male	25	Management Science	2	2
M3	Male	25	Business Administration	3	1
M4	Male	29	Business Administration	2	1
M5	Male	25	Agricultural Engineering	6	2
M6	Male	26	Vehicle Engineering	1	1
M7	Male	34	Organic Chemistry	5	3
F1	Female	28	Business Administration	3	9
F2	Female	30	Business Administration	1	7
F3	Female	26	Management Science	1	3
F4	Female	26	Business Administration	2	2
F5	Female	25	Business Administration	1	3
F6	Female	38	Business Administration	2	11
F7	Female	26	Business Administration	1	5
F8	Female	28	Vehicle Engineering	1	4
F9	Female	30	Food Science and Engineering	2	1
F10	Female	31	Organic Chemistry	3	1
F11	Female	30	Management Science	1	1

### 3.4. Analysis

#### 3.4.1. Open coding

The purpose of open coding is to abstract and conceptualize raw data. By carefully reading and analyzing the content of each sentence word-by-word, the underlying values and meanings were labeled with concepts. Through repeated comparisons, original statements that described the same concept and appeared more than three times were conceptualized and categorized. Eventually, they were summarized into 34 initial concepts and 17 initial categories, as shown in Table 2.

**Table 2:** Open coding examples.

Interviewee	Initial account	Concept	Category
M3	In our field, the number of Chinese journals is relatively small, while the number of English journals is comparatively higher.	Number of journals	Number Indicator
F4	We seldom read Chinese journals, as we primarily rely on English papers to keep up with the latest research. The number of cutting-edge studies that can be referenced from Chinese journals is relatively limited.	Number of papers	
F1	Generally, journals with high impact factors are English journals. To publish high-quality papers, it is necessary to publish in English journals.	Journal impact factor	Impact factor
M6	In the field of academia, high-impact scholars are determined by the number of citations. Only by publishing papers in English can one obtain a higher number of citations.	Number of citations	
M1	Professional SCI journals are primarily in English and have relatively higher impact factors, making them widely read by audiences.	Audience	
M5	It costs about 3,000 RMB to have a 7,000–8,000 word paper proofread once, and the price doubles for unlimited revisions.	Proofreading fee	Monetary cost
M9	Chinese journals require authors to pay page charges, which can range from 3,000 to 4,000 RMB per article. However, many English journals do not require any page charges.	Publication fee	
M2	The logical structure of English papers feels more rigorous, and I want to challenge myself by writing in English.	Challenging yourself	Spiritual rewards
F3	Writing English papers can help improve one’s English skills, as English is one of the most widely used languages in the world.	Self-improvement	
M5	think that the academic ethics and standards of English-language journals are high, and publishing in such journals can better prove my academic abilities.	Self-recognition	

#### 3.4.2. Axial coding

Building on the open coding, we differentiated between core and subcategories by repeatedly categorizing and adjusting similar categories based on their logical relationships and sequencing. Through this process, four core categories and nine subcategories were identified (see Table 3).

**Table 3:** Axial coding results.

Category (17)	Theme (9)	Dimension (4)
Number Indicator	Journal Indicator Attributes	Journal Attributes
Impact factor		
Academic Normativity	Journal Specification Attributes	
Content standardization		
Effectiveness of feedback	Journal Feedback Attributes	
Timeliness of feedback		
Professionalism of feedback		
Monetary cost	Perceived Cost Value	Perceived Value
Time cost		
Emotional cost		
Spiritual rewards	Perceived Return Value	
Material Return		
Social prestige	Perceived Connectivity Value	
International exchange		
External Stimulation	Environmental Context	Contextual factors
Internal Rendering	Physical Context	
Intention to choose English/Chinese	Intention to choose a language	Language choice

#### 3.4.3. Integration

**Figure 1:**
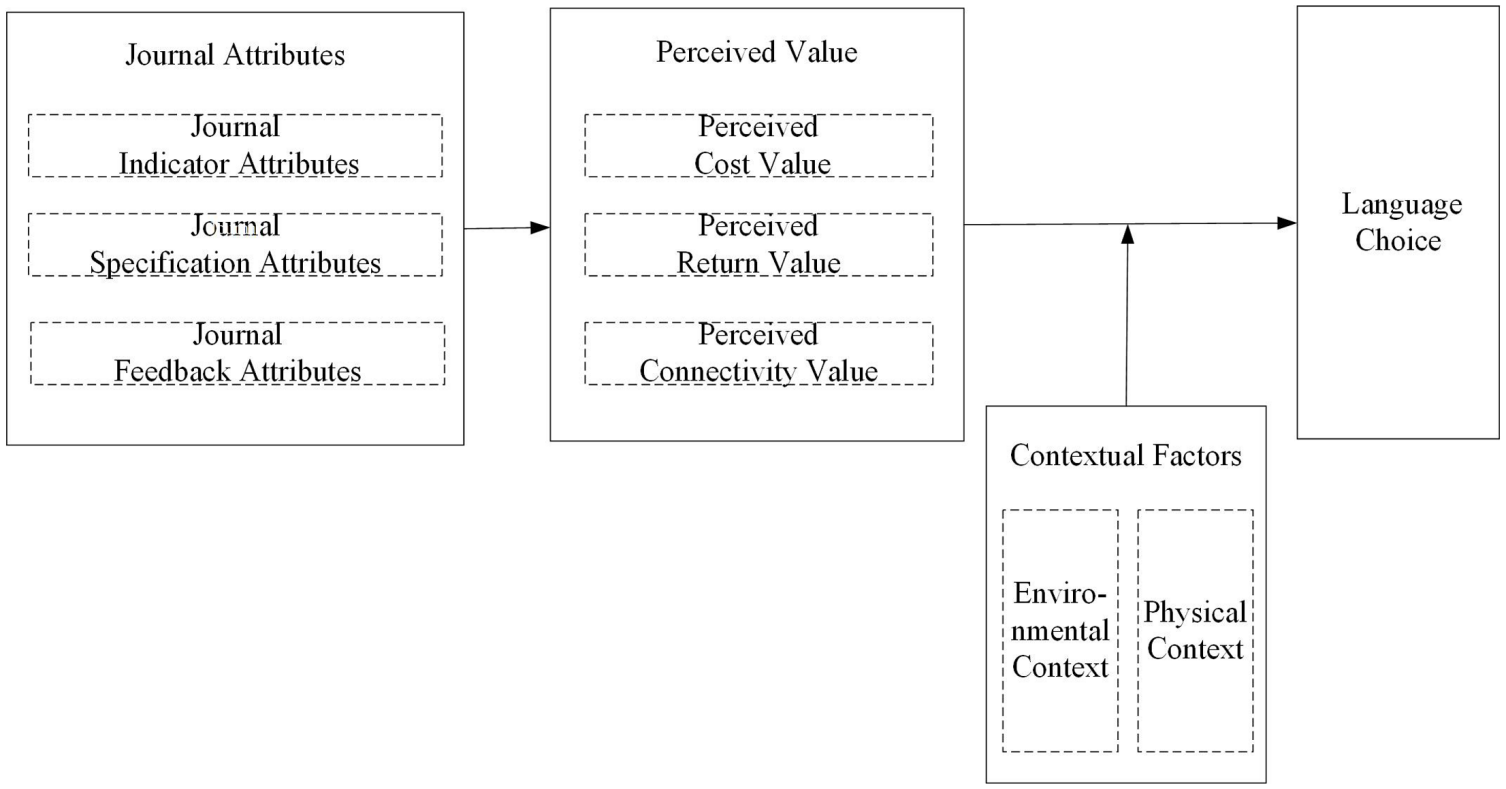
Conceptual framework.

Integration involves the consolidation of categories around a central category, and the subsequent refinement and streamlining of the resulting theoretical framework (Corbin et al., 2008, p. 351). Based on these dimensions, it can be summarized that journal attributes significantly influence researchers’ perceived value and academic language choice. Perceived value has a significant impact on researchers’ language choice in their academic papers. Contextual factors play a moderating role in the process by which journal attributes influence researchers’ language choice. Based on this “storyline,” this study developed an innovative model of the impact of journal attributes on researchers’ language choice in their academic papers, as shown in Figure 1.

To ensure the systematic construction of the theory, a theoretical saturation test was conducted on the coding results to determine that there were no new categories that could be explored in the analysis of the 15 interview records. Furthermore, grounded analysis was conducted on three randomly selected interview materials, which did not produce any new concepts or associative relationships; thus, all steps were ultimately terminated, and it was confirmed that our model of the impact of journal attributes on researchers’ language choice had reached saturation.

## 4. Results and discussion

The results of this study demonstrate the mechanism of language selection by Chinese scholars for their academic papers. The results show that: first, journal attributes influence researchers’ perceived value. Second, perceived value affects language choice. Third, contextual factors play a moderating role. The main findings and related discussions are now presented in more detail.

### 4.1. Journal attributes

Based on an analysis of the interviews and relevant literature, this article defines journal attributes as the inherent characteristics of journals as knowledge dissemination media (Lei and Jiang, 2019), which include journal indicators, journal specifications, and journal feedback information.

Journal indicators mainly include quantitative and impact indicators. Quantitative indicators refer to objective quantitative data on journal and article output. Impact indicators reflect the dissemination status of journal information, i.e., the degree to which the journal is read, referenced, cited, and used by readers after publication, including journal impact factor, citation frequency, reference quantity, and audience. Some studies have shown that the most important factors influencing authors’ choices of different journals are journal reputation, audience, and journal impact factor (Rowlands and Nicholas, 2005; Tenopir et al., 2016). Publications in English are frequently associated with utilitarian goals, such as gaining international recognition and reputation (Burgess et al., 2014; Muresan and Pérez-Llantada, 2014; Rowley et al., 2020) and obtaining monetary rewards (Lillis and Curry, 2010; Hanauer and Englander, 2011). This ties well with our findings, wherein journal indicators including journal reputation, audience, and journal impact factor seem to revolve around the perceived return value of scholarly publishing, as six interviewees mentioned: “the quality recognition of English papers in the evaluation and assessment is relatively high, which can provide more opportunities for salary increases.” Articles can be published in different languages to cater to various audience groups and characteristics, including audience type (Tenopir et al., 2016), number (Rowley et al., 2017), and geographic region (Ganasegeran et al., 2020). Utilizing different languages and journals of varying prestige can result in precise dissemination to a specific audience, which can be rewarded with local prestige and recognition as well as better communication. Seven interviewees believed that journal indicators have an impact on the perceived connectivity value. For example “publishing papers in English can internationalize one’s scientific research achievements, attract attention from peers worldwide, and gain recognition from more people.”

Writing research articles requires not only academic normativity but also writing skills. However, for non-native English-speaking scholars, writing research articles in English requires more time and effort. Accordingly, publishing in English may place an additional burden on them (Tardy, 2004; Shin et al., 2014). The process of publishing a paper includes the stages of submission, response, and revision; if a paper is submitted in English, each stage requires English language skills. Thus, acquiring the skills needed to publish in English implies additional time and energy costs. This is also supported by our analysis of the interviews, such as “having to think repeatedly about English articles, which can be quite stressful.” When non-native English-speaking scholars successfully publish in English, they perceive return values, such as satisfaction (Hanauer and Englander, 2011) and accomplishment in overcoming hurdles. As one interviewee expressed, “the logical mode of English articles feels more rigorous, so I want to challenge myself.”

Journal feedback attributes include the effectiveness, timeliness, and professionalism of feedback. The effectiveness of feedback was evaluated based on the quality of replies, review comments, and related services provided by the journal. The effectiveness of reviewers’ comments and the helpfulness of editors’ replies are significant deciding factors (Rowley et al., 2020). The timeliness of feedback was assessed based on the overall time from submission to publication or on specific subprocesses. A shorter turnaround time is a motivating factor in journal selection (Jamali et al., 2014; Weckowska et al., 2017). In line with previous studies, nine interviewees recognized the impact of journal feedback information attributes on perceived cost value. For example, “the review cycle for Chinese papers is too long, and I thought about translating it into English and submitting it to an English-language journal.” The professionalism of feedback was measured by the authority of the editor. Fair and professional review comments can greatly improve the quality of a paper (Lee et al., 2020). Even if a paper is ultimately rejected, the time invested will not be completely wasted, as the return value outweighs the value cost (Poelmans and Sandra, 2015). As one interviewee mentioned, “external reviewers are experts in the field and can provide better evaluations, and this kind of evaluation can benefit me a lot.” Researchers can improve the quality and impact of their papers by incorporating and adapting to the feedback they have received. By drawing on this feedback, researchers can refine their methods and the way they present their results. This iterative process of improvement not only enhances the overall quality of papers but also adds value to the perception of connectivity within the academic community.

In general, the analysis results indicate that the indicators, specifications, and feedback attributes of journals affect authors’ perceived cost value, perceived return value, and perceived connectivity value.

### 4.2. Perceived value

Perceived value has been studied extensively in the field of marketing, and there is a lot of research on the influence of perceived value on willingness to pay; however, little research has been conducted on the intention to choose a publication language. From the analysis of the coding results, perceived value can be defined as the subjective evaluation or cognition of the balance between the benefits perceived by researchers and the costs they pay when obtaining services, including perceived cost value, perceived return value, and perceived connectivity value (Sweeney and Soutar, 2001).

Perceived cost value includes monetary, time, and emotional costs (Strader and Shaw, 1999; Morewedge et al., 2007; Benazi et al., 2015). According to the interview results, six interviewees believed that perceived cost value has a significant impact on their language selection preference for publishing papers, such as “writing an English paper requires more time and effort, so I prefer to write in Chinese.”

Driven by their pursuit of academic job prospects, promotions, and tenure, they meticulously select the best-fitting journals (Conley et al., 2011). Seven interviewees acknowledged that perceived return value has a significant impact on their language selection preference, such as “to meet promotion and assessment requirements within a limited time, I would choose to publish in Chinese.”

Perceived connectivity value includes social reputation and international communication. Five interviewees believed that perceived connectivity value has an impact on their language selection preference, such as “sharing my research results with international scholars will get a more comprehensive evaluation, so I prefer to publish in English.”

The results indicate that perceived value mediates the relationship between journal attributes and language choice for publishing. No previous studies have examined the mediating effect of perceived value on article language choice. These findings thus extend our knowledge of the mechanisms underlying publishing language choice.

### 4.3. Contextual factors

Context, also known as situation or circumstance, is related to the factors of the subject and the political, economic, legal, ideological, and physical environment in which they exist (Chang and Tian, 2020). In this study, contextual factors mainly included environmental and physical contexts. The environmental context refers to the policies, regulations, and systems of the country, research institutions, schools, and other factors that influence researchers’ choice of a particular language for their papers. Physical context refers to the sensory atmosphere created by the language used by the people around researchers during the journal selection process.

The reviewed studies provide evidence that publication requirements and regulations prescribed by government agencies or affiliated institutions affect authors’ journal selection (Peekhaus and Proferes, 2016; Nelson and Eggett, 2017). Nine interviewees believed that external stimuli as moderating variables played a role in the relationship between perceived value and language selection. For example, “My English level is not very good, but I prefer to publish in English to meet promotion requirements” (the relationship between perceived cost value and language selection); “I think publishing in English will be more helpful to me in the future, provided that I have already met the graduation requirements” (the relationship between perceived return value and language selection); “Although there is no mandatory requirement for the language of the thesis at the school, I do not want to spend too much effort on writing English papers, although I want to get recognition from more people” (the relationship between perceived connectivity value and language selection).

Based on the interview content, more than half of the participants believed that physical context played an important role in influencing their language choice for academic papers based on perceived value. For example, one participant stated, “Using the mother tongue to publish papers should save a lot of time and effort, but because none of the senior students in our lab write in Chinese, it has become a default that we all have to write in English. So, I am more willing to publish in English and not be an outlier” (relationship between perceived cost value and language selection). Another participant mentioned, “Writing papers in English can help with job promotions, but since all of my co-authors are Chinese and we communicate in Chinese rather than English, I am more willing to publish in Chinese” (relationship between perceived reward value and language selection). Another participant mentioned, “English papers not only have high international recognition, but also our peers evaluate academic abilities based on the number of English papers published. Therefore, I am more willing to publish in English” (relationship between perceived connectivity value and language selection).

In summary, this study argues that situational factors play a moderating role in the influence of perceived value on researchers’ specific language choice for their academic papers.

## 5. Conclusion

Based on grounded theory, this paper has constructed concepts linking journal attributes, perceived value, contextual factors, and researchers’ language choice, and then proposed a theoretical model of the mechanism by which journal attributes affect researchers’ academic language choice. The model reveals the internal paths through which journal attributes affect perceived value, perceived value affects language selection, and contextual factors play a moderating role in the relationship between perceived value and academic language choice. In conclusion, the research findings are significant because they shed new light on the factors that influence Chinese researchers’ choice of publishing language from a perceived value perspective. Accordingly, this study provides suggestions for policymakers and journal operators when developing policies for language use and operating journals as follows.

(1) It is important to develop strategies to strengthen the influence of journal attributes. First, the journal should be disseminated through various marketing channels to increase its influence. The higher the influence of the journal, the higher the perceived value for researchers. Second, the scope of the journal’s content should be broadened and high-quality content should be selected to provide authoritative, professional, diverse, and in-depth material to readers (Poelmans and Sandra, 2015). Thirdly, the composition of the editorial team is crucial; it is important to invite experts with high reputations in their respective fields to join, which will have a “star effect” and enhance the trust of contributors in the journal, thus increasing the perceived value of the returns. (2) Strategies should also be developed to enhance researchers’ perceived value. First, services such as priority publications and discounted publication fees for high-quality papers should be provided to reduce the perceived cost value for researchers. Second, the editing and reviewing teams’ ability to provide timely and professional reviews can substantially improve the quality of published papers. Even if the waiting period for review is slightly longer, researchers still feel a sense of reward from the submission process. Third, providing free translation services that allow outstanding Chinese papers to be resubmitted abroad would provide researchers with a multilingual academic communication platform to enhance their perceived connectivity value. (3) Strategies are needed to enhance the impact of contextual factors. Some universities adopt a one-size-fits-all approach to improving the status and weight of Chinese academic papers in research achievement evaluation and title appointment policies in order to eliminate the “SCI-only supremacy” academic culture. This approach has given rise to a conflict between the preservation of native language integrity in academic discourse and the necessity for international scholarly exchange. Therefore, it is necessary to develop a fair evaluation system for both English and Chinese papers at the national and university levels to enhance the cultural self-confidence of non-native English-speaking researchers and raise awareness of research pluralism to realize sustainable academic development.

Although this study provides insights and references on the relationship between journal attributes and researchers’ language preferences, there are some limitations that need to be addressed. First, this study constructs a theoretical model based on a literature review and interview data but does not validate the model through empirical evidence. The reliability and validity of the impact model constructed based on grounded theory must be further tested. Future research should develop scales for each variable and conduct more detailed empirical studies to improve the universality of the theoretical research. Second, the number of interviewees in the study was limited. Although our analysis passed the theoretical saturation test, researchers from all disciplines and ages were not included. In future, researchers should collect more meaningful data to clarify the influence of the individual characteristics of researchers from different academic backgrounds and qualifications on their language preference for papers.

## Data availability statement

The original contributions presented in the study are included in the article/Supplementary material, further inquiries can be directed to the corresponding author.

## Ethics statement

Studies involving human participants were reviewed and approved by the Ethics Committee of School of Business and Management, Jilin University. All the participants provided written informed consent to participate in this study.

## Author contributions

JC performed the data analyses and wrote the manuscript. CQ helped perform the analyses through constructive discussion. ZW contributed to the implementation of the methods and manuscript preparation. All the authors contributed to the manuscript and approved the submitted version.

## Conflict of interest

The authors declare that the research was conducted in the absence of any commercial or financial relationships that could be construed as a potential conflict of interest.

## Publisher’s note

All claims expressed in this article are solely those of the authors and do not necessarily represent those of their affiliated organizations, or those of the publisher, the editors and the reviewers. Any product that may be evaluated in this article, or claim that may be made by its manufacturer, is not guaranteed or endorsed by the publisher.
