# Supplementary material for: Publishing in English or Chinese: a qualitative analysis of Chinese researchers’ academic language choice
Source: Front Psychol. 2023 Sep 20;14:1249857. doi: 10.3389/fpsyg.2023.1249857 (PMC10547893; doi:10.3389/fpsyg.2023.1249857)
Supplement: Supplementary file 1 [file Table_1.DOCX]

Supplementary Material

How do journal attributes affect the choice of language in academic papers?: A qualitative analysis on the willingness of Chinese researchers

Jing Cui, Changbo Qiu*, Zhigang Wang

*** Correspondence:** Changbo Qiu: qiucb@jlu.edu.cn

## Open coding results

| **Interviewee** | **Initial account** | **Concept** | **Category** |
| --- | --- | --- | --- |
| M3 | In our field, the number of Chinese journals is relatively small, while the number of English journals is comparatively higher. | Number of Journals | Number Indicator |
| F4 | We seldom read Chinese journals, as we primarily rely on English papers to keep up with the latest research. The number of cutting-edge studies that can be referenced from Chinese journals is relatively limited. | Number of papers |  |
| F1 | Generally, journals with high impact factors are English journals. To publish high-quality papers, it is necessary to publish in English journals. | Journal Impact Factor | Impact factor |
| M6 | In the field of academia, high-impact scholars are determined by the number of citations. Only by publishing papers in English can one obtain a higher number of citations. | Number of citations |  |
| M1 | Professional SCI journals are primarily in English and have relatively higher impact factors, making them widely read by audiences. | Audience |  |
| M7 | There have been many reports of academic misconduct in China recently, which makes us feel that English journals have higher standards for academic ethics. | Code of academic ethics | Academic Normativity |
| M5 | It seems difficult to publish papers in high-quality domestic journals if students publish papers under their own name or if they are not affiliated with prestigious universities like 211 universities in China | Fair and Just |  |
| F2 | There are significant differences in the logical expression and writing structure between Chinese and English. English literature follows a standardized logical thinking pattern, making it easier to read and write. | Normative model | Content standardization |
| F8 | Journal selection is based on the match between the research content and the preference of some foreign journals. There may not be differences in the theoretical and research content between domestic and foreign journals, but certain journals may have preferences. | Journal Preference |  |
| F9 | The comments given by external reviewers during the peer-review process are very helpful to me, and I appreciate that the editor-in-chief also reviews the manuscript and provides additional feedback. | Rigorous review of manuscripts | Effectiveness of feedback |
| M2 | It sounds like you received a very comprehensive set of reviewer comments that were very helpful in guiding your revisions. Taking the time to address all the comments in a thorough manner can greatly improve the quality of the manuscript and increase the chances of acceptance. | Detailed review comments |  |
| F11 | It seems that you find the peer review process for Chinese journals to be too long, and you had a paper rejected. You are considering translating the paper into English and submitting it to an English-language journal. | Refereeing period | Timeliness of feedback |
| F3 | I submit my articles to niche journals, and the external reviewers are experts in the field, which enables me to receive better evaluations. | Feedback Information Professional | Professionalism of feedback |
| M4 | Based on the composition of the editorial board and committee, if there are several top scholars, I have a lot of trust in the journal and believe that the review comments will be very authoritative. | Authoritative feedback |  |
| M5 | It costs about 3,000 RMB to have a 7,000-8,000 word paper proofread once, and the price doubles for unlimited revisions. | Proofreading fee | Monetary cost |
| M9 | Chinese journals require authors to pay page charges, which can range from 3000 to 4000 RMB per article. However, many English journals do not require any page charges. | Publication fee |  |
| F7 | To write a good English paper, I must give up other activities I enjoy and dedicate more time to writing. | Time cost | Time cost |
| F5 | Because English is not my native language, I worry about expressing myself inaccurately in writing. Additionally, I have to think through my English articles repeatedly, which can lead to a high level of anxiety. | Anxiety | Emotional cost |
| F8 | Having limited proficiency in English, I tend to be more cautious and careful when writing academic papers in English. | Caution |  |
| M2 | The logical structure of English papers feels more rigorous, and I want to challenge myself by writing in English. | Challenging yourself | Spiritual rewards |
| F3 | Writing English papers can help improve one's English skills, as English is one of the most widely used languages in the world. | Self-improvement |  |
| M5 | think that the academic ethics and standards of English-language journals are high, and publishing in such journals can better prove my academic abilities. | Self-recognition |  |
| F7 | Some universities have entry standards that require a minimum of two CSSCI papers, and I feel that the future demand for the number of Chinese papers will continue to increase. | Beneficial for Employment | Material Return |
| M7 | In the evaluation process, the quality of English papers is relatively highly recognized, and I feel that it can bring more opportunities for salary increase. | Compensation |  |
| M5 | Writing papers in English can help internationalize one's research results and gain recognition from peers worldwide. | Recognition by peers | Social prestige |
| M1 | English-language journals have higher international recognition, while Chinese-language journals have lower levels of attention. | International recognition |  |
| F10 | It helps to enhance personal, institutional, and research group academic status and better integrate into the entire foreign research community. | Integrating into the research community | International exchange |
| M3 | One of the papers I collaborated with my foreign advisor on had to be written in English | International collaboration |  |
| F9 | Publishing research papers in English can help me share my research ideas, innovations with my research field. | Peer communication |  |
| F5 | The policies requiring at least one high-quality paper in a Chinese journal. Therefore, I feel that it is also important to write good Chinese papers. | Policy guidance | External Stimulation |
| F6 | The promotion and evaluation system of the university places greater emphasis on English-language papers, which means that writing papers in English brings greater benefits. | Evaluation of Professional Qualifications |  |
| M2 | The graduation requirement of my school is to publish SCI papers, so in order to meet this requirement, I need to write English papers. | Graduation requirements |  |
| M5 | I feel that publishing in SCI and SSCI journals is in line with the trend. | Trend highlighting | Internal Rendering |
| F4 | I am familiar with the logical structure of English writing and have become accustomed to this pattern. I find it more comfortable and am more willing to write in English. | Choice willingness | Willingness to choose |
